# Supplementary material for: A randomized trial evaluating virus-specific effects of a combination probiotic in children with acute gastroenteritis
Source: Nat Commun. 2020 May 21;11:2533. doi: 10.1038/s41467-020-16308-3 (PMC7242434; doi:10.1038/s41467-020-16308-3)
Supplement: Supplementary file 1 — Supplementary Information [file 41467_2020_16308_MOESM1_ESM.pdf]

Supplementary Information

Manuscript Title: A Randomized Trial Evaluating Virus-Specific Effects of a Combination Probiotic in Children with Acute Gastroenteritis

Authors: Freedman et al.

Supplementary Table 1. Pre-study anticipated mean scores per pathogen group employed to conduct sample size estimations. They were based on best availability of published estimates in the literature[1] and the beneficial effects of probiotics.[2]

	Probiotic Group	Placebo Group
Pathogen Group		
Virus	10.6	13.6
Bacteria	14.0	15.0
Not identified	7.0	7.0
Specific Virus		
Rotavirus	10.5	16.0
Norovirus	12.0	14.0
Adenovirus	8.0	8.0

Supplementary Table 2. Listing of pathogens and frequencies.

**Enteropathogens Detected**	**Probiotics N=408**	**Placebo N=408**
Norovirus GI and/or GII, n (%)	99 (24.3)	116 (28.4)
Rotavirus, n (%)	115 (28.2)	79 (19.4)
Adenovirus, n (%)	50 (12.3)	44 (10.8)
C. difficile, n (%)	47 (11.5)	60 (14.7)
Salmonella spp., n (%)	11 (2.7)	7 (1.7)
Campylobacter spp., n (%)	8 (2.0)	3 (0.7)
Enterotoxigenic *Escherichia coli*, n (%)	2 (0.5)	8 (2.0)
Cryptosporidium, n (%)	6 (1.5)	3 (0.7)
C. difficile, n (%) (when restricted to > 2 years of age)	3 (0.7)	6 (1.5)
Shigella spp., n (%)	2 (0.5)	3 (0.7)
Shiga toxin-producing *E. coli*, n (%)	4 (1.0)	0 (0)
Giardia, n (%)	1 (0.2)	0 (0)
E. coli O157, n (%)	0 (0)	0 (0)

Supplemental Table 3. Clinical characteristics by pathogen group.

**Characteristics**	**Negative** N=299	**Virus Only** N=451	**Bacteria Only** N=37	**Virus/bacteria Co-Detection** N=19	**Other[a]** N=10
Median age (IQR), mo	13.0 (8.0, 24.0)	16.0 (10.0, 24.0)	21.0 (11.0, 33.5)	17.0 (11.0, 25.0)	24.5 (20.5, 32.3)
Male sex – no. (%)	171 (57.2)	256 (56.8)	20 (54.1)	11 (57.9)	6 (60.0)
Weight (IQR) — kg; median (IQR)	10.3 (8.6, 13.0)	10.7 (9.2, 12.5)	11.5 (8.8, 14.8)	11.0 (9.2, 12.2)	12.8 (11.3, 13.9)
Exclusive breast fed – no. (%)	21 (7.0)	27 (6.0)	2 (5.4)	1 (5.3)	0 (0)
Received antibiotics in previous 14 days — no. (%)	42 (14.0)	58 (12.9)	3 (8.1)	3 (15.8)	2 (20.0)
Received rotavirus vaccine — no. (%)	167 (55.9)	194 (43.0)	16 (43.2)	12 (63.2)	2 (20.0)
Duration of illness (IQR) — hr; median (IQR)†	41.4 (26.5, 57.1)	44.9 (28.5, 60.4)	31.3 (23.5, 47.5)	51.0 (35.0, 59.7)	43.2 (28.9, 61.6)
Baseline Modified Vesikari Scale Score - median (IQR)‡	10.0 (8.0, 12.0)	12.0 (10.0, 13.0)	10.0 (8.0, 12.0)	12.0 (10.0, 13.0)	12.0 (10.0, 15.0)
Vomiting — no. (%)	178 (59.5)	406 (90.0)	18 (48.6)	15 (78.9)	7 (70.0)
No. of vomiting episodes in preceding 24 hr - median (IQR)§	3 (1, 5)	4 (2, 7)	2 (1, 5)	4 (2, 16)	4 (1, 8)
No. of diarrhea episodes in preceding 24 hr - median (IQR)	5 (3, 7)	5 (3, 8)	6 (4, 13)	9 (5, 10)	4 (3, 9)
Febrile — no. (%)¶	142 (47.5)	181 (40.1)	23 (62.2)	8 (42.1)	7 (70.0)

Clinical Dehydration Scale Score – median (IQR)‖	0 (0, 1)	1 (0, 2)	1 (0, 2)	1 (0, 3)	0 (0, 1)
Received ondansetron at index visit — no. (%)	37 (12.4)	135 (29.9)	2 (5.4)	5 (26.3)	0 (0)
Received antibiotics at index visit / recommended at discharge— no	9 (3.0)	6 (1.3)	0 (0)	0 (0)	0 (0)
Received intravenous rehydration at index visit — no (%)	13 (4.3)	47 (10.4)	3 (8.1)	3 (15.8)	1 (10.0)
Admitted to hospital at index visit — no. (%)	7 (2.3)	13 (2.9)	0 (0)	0 (0)	0 (0)

[a] This group included parasite only=6, virus/parasite co-detection=4.

Supplementary Table 4. Weighted linear regression analysis with Modified Vesikari Scale score as dependent variable including *a priori* identified covariates and interaction terms for treatment allocation and pathogen groups. The reported P-values are two-sided.

	**Complete Cases (N=433)**		**Imputation Dataset (N=816)**	
	**Mean Difference (95%CI)**	**P-Value**	**Mean Difference (95%CI)**	**P-Value**
Probiotics, Yes	-0.015 (-1.128, 1.098)	0.979	-0.385 (-1.276, 0.506)	0.397
Sex, Female	-0.365 (-1.079, 0.349)	0.317	0.065 (-0.537, 0.666)	0.833
Age, Months	-0.054 (-0.083, -0.025)	0.0003	-0.077 (-0.102, -0.051)	5.02×10^{-9}
Antibiotics in the Past 14 days, Yes	-0.067 (-0.999, 0.865)	0.888	-0.411 (-1.277, 0.456)	0.353
Index Visit Modified Vesikari Scale Score	0.339 (0.198, 0.480)	2.35×10^{-6}	0.359 (0.247, 0.471)	3.68×10^{-10}
Admitted to Hospital, Yes	1.571 (-0.822, 3.965)	0.198	1.201 (-0.400, 2.801)	0.141
**Infectious agent**				
Virus only	0.212 (-0.859, 1.282)	0.698	0.029 (-0.826, 0.883)	0.948
Bacteria only	2.179 (0.378, 3.980)	0.018	1.792 (-0.160, 3.745)	0.072
Virus/Bacteria Co-Detection	2.864 (-1.514, 7.243)	0.200	0.884 (-2.932, 4.699)	0.650
Parasite ± Virus Co-Detection	-2.416 (-9.077, 4.246)	0.477	-1.844 (-7.600, 3.912)	0.530
No Pathogen	Reference			
**Interaction terms**				
[probiotics] * [other]	0.981 (-6.782, 8.744)	0.804	4.616 (-2.289, 11.522)	0.190
[probiotics] * [co-detection]	-2.277 (-7.674, 3.121)	0.408	-0.151 (-4.913, 4.610)	0.950
[probiotics] * [bacteria only]	-0.210 (-3.103, 2.683)	0.887	2.040 (-1.023, 5.102)	0.192
[probiotics] * [virus only]	0.572 (-0.908, 2.052)	0.449	0.687 (-0.525, 1.900)	0.267

Supplementary Table 5. Weighted linear regression with Modified Vesikari Scale score as dependent variable including key covariates; with interaction terms removed (i.e. final model as the interaction terms were not insignificant when included in the model – Supplementary Table 3). The reported P-values are two-sided.

	**Complete Cases (N=433)**		**Imputation Dataset (N=816)**	
	**Mean Difference (95%CI)**	**P-Value**	**Mean Difference (95%CI)**	**P-Value**
Probiotics, Yes	0.231 (-0.461, 0.924)	0.512	0.077 (-0.506, 0.660)	0.795
Sex, Female	-0.363 (-1.075, 0.350)	0.319	0.043 (-0.558, 0.645)	0.888
Age, Months	-0.055 (-0.084, -0.026)	0.0002	-0.078 (-0.104, -0.053)	1.82×10^{-9}
Antibiotics in the Past 14 days, Yes	-0.077 (-1.009, 0.854)	0.871	-0.418 (-1.286, 0.449)	0.345
Index Visit Modified Vesikari Scale Score	0.336 (0.197, 0.476)	2.21×10^{-6}	0.365 (0.253, 0.477)	1.81×10^{-10}
Admitted to Hospital, Yes	1.471 (-0.906, 3.848)	0.225	1.076 (-0.518, 2.671)	0.186
**Infectious agent**				
Virus only	0.549 (-0.294, 1.393)	0.202	0.312 (-0.353, 0.976)	0.358
Bacteria only	2.246 (0.553, 3.940)	0.009	2.727 (1.256, 4.199)	0.0003
Virus/Bacteria Co-Detection	1.085 (-1.099, 3.268)	0.330	0.474 (-1.553, 2.501)	0.647
Parasite ± Virus Co-Detection	-1.961 (-6.317, 2.394)	0.377	1.201 (-1.556, 3.958)	0.393
No Pathogen	Reference			

Supplementary Table 6. Subgroup linear regression model including only **pathogen negative participants** with Modified Vesikari Scale score as the dependent variable including key covariates. The reported P-values are two-sided.

	**Complete Cases (N=130)**		**Imputation Dataset (N=299)**	
	Mean difference (95%CI)	P-Value	Mean Difference (95%CI)	P-Value
Probiotics, Yes	-0.149 (-1.253, 0.955)	0.791	-0.398 (-1.306, 0.511)	0.391
Sex, Female	0.310 (-0.897, 1.517)	0.615	0.461 (-0.486, 1.409)	0.340
Age, Months	-0.061 (-0.103, -0.019)	0.004	-0.103 (-0.143, -0.063)	3.71×10^{-7}
Antibiotics in the Past 14 days, Yes	0.654 (-1.031, 2.339)	0.447	0.063 (-1.271, 1.397)	0.926
Index Visit Modified Vesikari Scale Score	0.299 (0.098, 0.500)	0.004	0.367 (0.205, 0.530)	9.00×10^{-6}
Admitted to Hospital, Yes	2.387 (-1.233, 6.007)	0.196	1.389 (-1.422, 4.199)	0.333

Supplementary Table 7. Subgroup linear regression model including only **bacteria positive** participants with Modified Vesikari Scale score as the dependent variable including key covariates**.** The reported P-values are two-sided.

	**Complete Cases (N=23)**		**Imputation Dataset (N=37)**	
	Mean Difference (95%CI)	P-Value	Mean Difference (95%CI)	P-Value
Probiotics, Yes	-0.784 (-3.619, 2.051)	0.588	-0.002 (-3.013, 3.01)	0.999
Sex, Female	-1.931 (-4.665, 0.804)	0.166	-2.579 (-5.272, 0.114)	0.061
Age, Months	-0.098 (-0.224, 0.029)	0.130	-0.122 (-0.233, -0.011)	0.031
Antibiotics in the Past 14 days, Yes	-0.592 (-4.505, 3.321)	0.767	-1.552 (-6.252, 3.148)	0.517
Index Visit Modified Vesikari Scale Score	0.630 (0.003, 1.258)	0.049	0.554 (0.012, 1.095)	0.045
Admitted to Hospital, Yes	N/A		N/A	

N/A, not able to estimate as 0 case was in the cell of "Admitted to Hospital, Yes".

Supplementary Table 8. Subgroup linear regression model including only **virus positive** participants with Modified Vesikari Scale score as the dependent variable including key covariates. The reported P-values are two-sided.

	**Complete Cases (N=264)**		**Imputation Dataset (N=451)**	
	Mean Difference (95%CI)	P-Value	Mean Difference (95%CI)	P-Value
Probiotics, Yes	0.538 (-0.446, 1.521)	0.284	0.224 (-0.609, 1.056)	0.599
Sex, Female	-0.885 (-1.868, 0.097)	0.077	-0.128 (-0.972, 0.715)	0.765
Age, Months	-0.04 (-0.085, 0.004)	0.076	-0.055 (-0.094, -0.016)	0.006
Antibiotics in the Past 14 days, Yes	-0.535 (-1.917, 0.847)	0.448	-0.357 (-1.597, 0.883)	0.573
Index Visit Modified Vesikari Scale Score	0.388 (0.171, 0.604)	4.54×10^{-4}	0.359 (0.182, 0.537)	7.40×10^{-5}
Admitted to Hospital, Yes	0.732 (-2.425, 3.889)	0.650	0.687 (-1.964, 3.338)	0.611

Supplementary Table 9. Subgroup linear regression model including only **virus/bacteria co-detection** participants with Modified Vesikari Scale score as the dependent variable including key covariates. The reported P-values are two-sided.

	**Complete Cases (N=13)**		**Imputation Dataset (N=19)**	
	Mean Difference (95%CI)	P-Value	Mean Difference (95%CI)	P-Value
Probiotics, Yes	-2.272 (-9.055, 4.51)	0.511	0.212 (-4.927, 5.35)	0.936
Sex, Female	-0.539 (-8.305, 7.226)	0.892	-0.930 (-5.974, 4.115)	0.718
Age, Months	0.235 (-0.121, 0.591)	0.196	0.141 (-0.153, 0.435)	0.346
Antibiotics in the Past 14 days, Yes	4.773 (-2.045, 11.592)	0.170	-0.655 (-6.52, 5.209)	0.827
Index Visit Modified Vesikari Scale Score	-0.82 (-2.271, 0.630)	0.268	-0.631 (-1.826, 0.564)	0.301
Admitted to Hospital, Yes	N/A		N/A	

N/A, not able to estimate as 0 case was in the cell of "Admitted to Hospital, Yes".

Supplementary Table 10. Subgroup linear regression model including only **parasite and parasite/virus co-detection** participants with Modified Vesikari Scale score as the dependent variable including key covariates. The reported P-values are two-sided.

	**Complete Cases (N=3)**		**Imputation Dataset (N=10)**	
	Mean Difference (95%CI)	P-Value	Mean Difference (95%CI)	P-Value
Probiotics, Yes	N/A		4.417 (-0.509, 9.343)	0.079
Sex, Female	N/A		0.020 (-5.223, 5.263)	0.994
Age, Months	N/A		0.026 (-0.192, 0.244)	0.815
Antibiotics in the Past 14 days, Yes	N/A		-5.576 (-11.447, 0.294)	0.063
Index Visit Modified Vesikari Scale Score	N/A		0.939 (-0.03, 1.908)	0.057
Admitted to Hospital, Yes	N/A		N/A	

N/A, not able to estimate as 0 case was in the cell of "Admitted to Hospital, Yes".

Supplementary Table 11. Subgroup linear regression model including only **adenovirus virus** positive participants with Modified Vesikari Scale score as the dependent variable including key covariates. The reported P-values are two-sided.

	**Complete Cases (N=58)**		**Imputation Dataset (N=94)**	
	Mean Difference (95%CI)	P-Value	Mean Difference (95%CI)	P-Value
Probiotics, Yes	-0.073 (-1.906, 1.76)	0.938	-0.020 (-1.648, 1.608)	0.981
Sex, Female	-0.854 (-2.644, 0.936)	0.350	-0.905 (-2.546, 0.736)	0.280
Age, Months	-0.078 (-0.176, 0.019)	0.116	-0.075 (-0.155, 0.005)	0.066
Antibiotics in the Past 14 days, Yes	1.450 (-0.758, 3.657)	0.198	0.417 (-1.773, 2.607)	0.709
Index Visit Modified Vesikari Scale Score	-0.041 (-0.432, 0.35)	0.837	0.203 (-0.137, 0.543)	0.243
Admitted to Hospital, Yes	-1.629 (-6.929, 3.671)	0.547	-0.894 (-5.868, 4.079)	0.725

Supplementary Table 12. Subgroup linear regression model including only **isolated adenovirus virus** positive participants (i.e. excluding those with co-detection) with Modified Vesikari Scale score as the dependent variable including key covariates. The reported P-values are two-sided.

	Complete Cases (N=43)		Imputation Dataset (N=74)	
	Mean Difference (95%CI)	P-Value	Mean Difference (95%CI)	P-Value
Probiotics, Yes	0.177 (-1.973, 2.327)	0.872	-0.184 (-1.876, 1.507)	0.831
Sex, Female	-0.793 (-2.89, 1.304)	0.459	-0.363 (-2.075, 1.349)	0.678
Age, Months	-0.055 (-0.173, 0.062)	0.357	-0.092 (-0.176, -0.008)	0.031
Antibiotics in the Past 14 days, Yes	1.618 (-0.872, 4.109)	0.203	0.899 (-1.357, 3.154)	0.435
Index Visit Modified Vesikari Scale Score	-0.035 (-0.532, 0.463)	0.891	0.173 (-0.198, 0.543)	0.361
Admitted to Hospital, Yes	-1.369 (-6.868, 4.129)	0.625	-0.664 (-5.327, 4.000)	0.780

Supplementary Table 13. Subgroup linear regression model including only **norovirus positive** participants with Modified Vesikari Scale score as the dependent variable including key covariates. The reported P-values are two-sided.

	**Complete Cases (N=112)**		**Imputation Dataset (N=215)**	
	Mean Difference (95%CI)	P-Value	Mean Difference (95%CI)	P-Value
Probiotics, Yes	0.724 (-0.658, 2.106)	0.304	0.401 (-0.811, 1.612)	0.517
Sex, Female	-1.589 (-2.998, -0.180)	0.027	-0.371 (-1.618, 0.877)	0.560
Age, Months	-0.131 (-0.203, -0.059)	3.48×10^{-4}	-0.085 (-0.149, -0.02)	0.011
Antibiotics in the Past 14 days, Yes	-2.040 (-4.203, 0.123)	0.065	-0.778 (-2.614, 1.057)	0.406
Index Visit Modified Vesikari Scale Score	0.470 (0.130, 0.810)	0.007	0.452 (0.174, 0.730)	0.001
Admitted to Hospital, Yes	5.581 (-1.947, 13.11)	0.146	2.055 (-3.126, 7.236)	0.437

Supplementary Table 14. Subgroup linear regression model including only **isolated norovirus virus** positive participants (i.e. excluding those with co-detection) with Modified Vesikari Scale score as the dependent variable including key covariates. The reported P-values are two-sided.

	**Complete Cases (N=99)**		**Imputation Dataset (N=187)**	
	Mean Difference (95%CI)	P-Value	Mean Difference (95%CI)	P-Value
Probiotics, Yes	0.627 (-0.831, 2.085)	0.399	-0.071 (-1.318, 1.175)	0.910
Sex, Female	-1.855 (-3.327, -0.383)	0.014	-0.286 (-1.556, 0.984)	0.659
Age, Months	-0.137 (-0.213, -0.06)	4.58×10^{-4}	-0.119 (-0.186, -0.052)	0.001
Antibiotics in the Past 14 days, Yes	-1.301 (-3.731, 1.13)	0.294	-0.361 (-2.325, 1.604)	0.719
Index Visit Modified Vesikari Scale Score	0.471 (0.109, 0.833)	0.011	0.439 (0.156, 0.722)	0.002
Admitted to Hospital, Yes	4.716 (-2.913, 12.346)	0.226	2.628 (-3.357, 8.613)	0.389

Supplementary Table 15. Subgroup linear regression model including only **rotavirus positive** participants with Modified Vesikari Scale score as the dependent variable including key covariates. The reported P-values are two-sided.

	**Complete Cases (N=125)**		**Imputation Dataset (N=194)**	
	Mean Difference (95%CI)	P-Value	Mean Difference (95%CI)	P-Value
Probiotics, Yes	0.378 (-1.117, 1.873)	0.620	0.292 (-1.097, 1.681)	0.680
Sex, Female	-0.697 (-2.179, 0.784)	0.356	-0.210 (-1.573, 1.152)	0.762
Age, Months	-0.011 (-0.073, 0.051)	0.722	-0.024 (-0.083, 0.035)	0.429
Antibiotics in the Past 14 days, Yes	-1.643 (-3.694, 0.409)	0.117	-1.027 (-2.986, 0.932)	0.304
Index Visit Modified Vesikari Scale Score	0.403 (0.085, 0.721)	0.013	0.220 (-0.077, 0.517)	0.147
Admitted to Hospital, Yes	0.726 (-3.599, 5.05)	0.742	0.893 (-2.825, 4.611)	0.638

Supplementary Table 16. Subgroup linear regression model including only **isolated rotavirus virus** positive participants (i.e. excluding those with co-detection) with Modified Vesikari Scale score as the dependent variable including key covariates. The reported P-values are two-sided.

	**Complete Cases (N=105)**		**Imputation Dataset (N=164)**	
	Mean Difference (95%CI)	P-Value	Mean Difference (95%CI)	P-Value
Probiotics, Yes	0.472 (-1.221, 2.166)	0.585	0.259 (-1.25, 1.767)	0.737
Sex, Female	-0.726 (-2.415, 0.963)	0.399	0.103 (-1.389, 1.594)	0.892
Age, Months	-0.002 (-0.072, 0.068)	0.953	-0.033 (-0.098, 0.031)	0.308
Antibiotics in the Past 14 days, Yes	-1.74 (-4.143, 0.664)	0.156	-1.180 (-3.451, 1.09)	0.308
Index Visit Modified Vesikari Scale Score	0.361 (0.004, 0.717)	0.047	0.210 (-0.108, 0.527)	0.196
Admitted to Hospital, Yes	0.767 (-3.773, 5.307)	0.741	0.885 (-3.153, 4.922)	0.668

Supplementary Table 17. Subgroup linear regression model including only ***Campylobacter* spp.** positive participants with Modified Vesikari Scale score as the dependent variable including key covariates. The reported P-values are two-sided.

	**Complete Cases (N=8)**		**Imputation Dataset (N=11)**	
	Mean Difference (95%CI)	P-Value	Mean Difference (95%CI)	P-Value
Probiotics, Yes	-1.212 (-3.282, 0.859)	0.251	2.992 (-3.055, 9.04)	0.332
Sex, Female	-1.2 (-3.45, 1.049)	0.296	-7.298 (-13.49, -1.106)	0.021
Age, Months	0.128 (0, 0.255)	0.050	0.192 (-0.188, 0.572)	0.322
Antibiotics in the Past 14 days, Yes	4.229 (1.427, 7.03)	0.003	-2.17 (-10.935, 6.595)	0.627
Index Visit Modified Vesikari Scale Score	0.125 (-0.313, 0.563)	0.577	-0.36 (-1.582, 0.861)	0.563
Admitted to Hospital, Yes	-1.212 (-3.282, 0.859)	0.251	2.992 (-3.055, 9.04)	0.332

Supplementary Table 18. Clinical characteristics by groups of participants who did and did not submit all three stool specimens.

**Characteristics**	**No**<br>**N=668**	**Yes**<br>**N=148**
Age mo, median (IQR)	16.0 (10.0, 24.8)	14.0 (9.3, 23.0)
Male sex – no. (%)	380 (43)	84 (57)
Weight median (IQR) — kg	10.7 (9.0, 12.9)	10.3 (8.8, 12.5)
Exclusive breast fed – no. (%)	42 (6)	9 (6)
Received antibiotics in previous 14 days — no. (%)	88 (13)	20 (14)
Received rotavirus vaccine — no. (%)		
Yes	308 (46)	83 (56)
No	191 (29)	34 (23)
Unsure	169 (25)	31 (21)
Duration of illness median (IQR) — hr†	43.5 (27.2, 58.8)	43.0 (27.5, 57.3)
Baseline Modified Vesikari Scale Score – mean (SD)‡	11.0 (2.8)	11.2 (2.5)
Vomiting — no. (%)	504 (75)	120 (81)
No. of vomiting episodes in preceding 24 hr – median (IQR)§	2 (0, 5)	3 (0, 6)
No. of diarrhea episodes in preceding 24 hr – median (IQR)	5 (3, 8)	5 (4, 9)
Febrile — no. (%)¶	295 (44)	66 (45)
Clinical Dehydration Scale Score – median (IQR)‖	1 (0, 2)	1 (0, 2)
Received ondansetron at index visit — no. (%)	140 (21)	39 (26)
Received antibiotics at index visit / recommended at discharge— no. (%)	12 (2)	3 (2)
Received intravenous rehydration at index visit — no. (%)	53 (8)	14 (10)
Admitted to hospital at index visit — no. (%)	18 (3)	2 (1)

† This variable was defined according to the duration of vomiting or the duration of diarrhea before enrollment, which- ever was greater
‡ Scores on the modified Vesikari scale range from 0 to 20, with higher scores indicating greater disease severity
¶ Febrile was defined as a documented adjusted rectal temperature of at least 38.0°C.
‖ Scores on the clinical dehydration scale range from 0 to 8, with higher scores indicating more severe dehydration.

Supplementary Table 19. Sub-group analyses based on participant age and pathogen detected.

	Probiotics		Placebo			
	**N**	**Mean (SD)**	**N**	**Mean (SD)**	**Mean difference (95%CI)**	**P-Value†**
**Test Negative**						
**Overall**	139	5.08 (4.1)	160	5.49 (4.4)	-0.41 (-1.38, 0.56)	0.41
**<1.0 yr**	57	6.73 (4.5)	64	6.77 (4.5)	-0.05 (-1.67, 1.57)	0.95
**1.0 yr to < 2.0 yr**	44	4.48 (3.7)	55	4.60 (4.2)	-0.12 (-1.73, 1.48)	0.88
**2.0 yr to < 3.0 yr**	21	3.05 (3.1)	26	5.44 (4.0)	-2.40 (-4.49, -2.98)	0.03
**3.0 yr to 4.0 yr**	17	3.67 (2.7)	15	3.40 (3.4)	0.27 (-1.87, 2.42)	0.80
**Virus Only**						
**Overall**	232	6.27 (4.7)	219	6.01 (4.4)	0.26 (-0.59, 1.12)	0.55
**<1.0 yr**	64	6.97 (4.8)	77	6.94 (4.2)	0.03 (-1.50, 1.56)	0.97
**1.0 yr to < 2.0 yr**	105	6.10 (4.7)	92	5.67 (4.4)	0.43 (-0.86, 1.72)	0.51
**2.0 yr to < 3.0 yr**	36	5.49 (4.9)	32	5.46 (4.5)	0.04 (-2.21, 2.28)	0.98
**3.0 yr to 4.0 yr**	27	6.30 (4.4)	18	4.71 (4.3)	1.58 (-1.02, 4.20)	0.24
**Bacteria Only**						
**Overall**	17	9.36 (5.3)	20	6.37 (4.2)	2.99 (-0.16, 6.08)	0.06
**<1.0 yr**	7	9.85 (6.0)	3	11.88 (4.5)	-2.03 (-9.834, 5.77)	0.61
**1.0 yr to < 2.0 yr**	6	9.52 (6.21)	3	7.00 (4.6)	2.52 (-5.49, 10.53)	0.54
**2.0 yr to < 3.0 yr**	3	9.00 (3.5)	8	4.24 (1.9)	4.76 (1.66, 7.87)	0.003
**3.0 yr to 4.0 yr**	1	6.00 (N/A)	6	6.13 (4.3)	-0.13 (-9.21, 8.96)	0.98
**Virus/Bacteria Co-Detection**						
**Overall**	13	6.21 (4.4)	6	6.39 (5.9)	-0.18 (-5.00, 4.64)	0.94
**<1.0 yr**	4	4.39 (5.1)	3	6.33 (8.5)	-1.95 (-12.02, 8.13)	0.71
**1.0 yr to < 2.0 yr**	4	5.61 (3.8)	1	2.15 (N/A)	3.46 (-4.94, 11.87)	0.42
**2.0 yr to < 3.0 yr**	5	8.15 (4.3)	2	8.60 (0.9)	-0.45 (-7.23, 6.33)	0.90
**3.0 yr to 4.0 yr**	0	N/A	0	N/A	N/A	N/A
**Other ***						
**Overall**	7	7.87 (5.7)	3	3.83 (1.4)	4.04 (-0.73, 8.81)	0.10
**<1.0 yr**	1	4.00 (N/A)	0	N/A	N/A	N/A
**1.0 yr to < 2.0 yr**	2	7.15 (10.1)	1	5.50 (N/A)	1.65 (-22.84, 26.14)	0.90
**2.0 yr to < 3.0 yr**	3	9.93 (5.4)	1	3.00 (N/A)	6.93 (-5.48, 19.35)	0.27

**3.0 yr to 4.0 yr**	1	7.00 (N/A)	1	3.00 (N/A)	4.00 (N/A)	N/A

*Parasite only and virus-parasite co-detection

† Statistical testing was performed using the T-test. Reported P-values reported are two-sided and unadjusted for multiple comparison.

A P-value < 0.0023 is considered statistically significant after adjustment for multiple comparison using Bonferroni method (n=22).

Supplementary Table 20. Sub-group analyses based on breast-feeding status and pathogen detected.

	Probiotics		Placebo			
	**N**	**Mean (SD)**	**N**	**Mean (SD)**	**Mean difference (95%CI)**	**P-Value†**
**Test Negative**						
**Overall**	139	5.08 (4.1)	160	5.49 (4.4)	-0.41 (-1.38, 0.56)	0.41
**Exclusive breastfed**	6	3.69 (5.3)	15	7.36 (5.3)	-3.67 (-8.92, 1.58)	0.17
**Not exclusive breastfed**	133	5.15 (4.0)	145	5.30 (4.2)	-0.15 (-1.14, 0.83)	0.76
**Virus Only**						
**Overall**	232	6.27 (4.7)	219	6.01 (4.4)	0.26 (-0.59, 1.12)	0.55
**Exclusive breastfed**	14	6.40 (4.2)	13	6.71 (4.0)	-0.31 (-3.44, 2.82)	0.85
**Not exclusive breastfed**	218	6.26 (4.8)	206	5.96 (4.4)	0.30 (-0.59, 1.18)	0.51
**Bacteria Only**						
**Overall**	17	9.36 (5.3)	20	6.37 (4.2)	2.99 (-0.16, 6.08)	0.06
**Exclusive breastfed**	1	13.00 (N/A)	1	17.00 (N/A)	N/A	N/A
**Not exclusive breastfed**	16	9.13 (5.4)	19	5.88 (3.6)	3.25 (0.10, 6.41)	0.04
**Virus/Bacteria Co-Detection**						
**Overall**	13	6.21 (4.4)	6	6.39 (5.9)	-0.18 (-5.00, 4.64)	0.94
**Exclusive breastfed**	1	8.00 (N/A)	0	N/A	N/A	N/A
**Not exclusive breastfed**	12	6.06 (4.6)	6	6.39 (5.9)	-0.33 (-5.79, 5.13)	0.91
**Other ***						
**Overall**	7	7.87 (5.7)	3	3.83 (1.4)	4.04 (-0.73, 8.81)	0.10
**Exclusive breastfed**	0	N/A	0	N/A	N/A	N/A
**Not exclusive breastfed**	7	7.87 (5.7)	3	3.83 (1.4)	4.04 (-0.73, 8.81)	0.10

*Parasite only and virus-parasite co-detection

† Statistical testing was performed using the T-test. Reported P-values reported are two-sided and unadjusted for multiple comparison.

A P-value < 0.004 is considered statistically significant after adjustment for multiple comparison using Bonferroni method (n=12).

References

1. Rasanen S, Lappalainen S, Salminen M, Huhti L, Vesikari T. Noroviruses in children seen in a hospital for acute gastroenteritis in Finland. Eur J Pediatr 2011;170:1413-8.
2. Huang YF, Liu PY, Chen YY, et al. Three-combination probiotics therapy in children with salmonella and rotavirus gastroenteritis. J Clin Gastroenterol 2014;48:37-42.
