# Supplementary material for: A randomized trial evaluating virus-specific effects of a combination probiotic in children with acute gastroenteritis
Source: Nat Commun. 2020 May 21;11:2533. doi: 10.1038/s41467-020-16308-3 (PMC7242434; doi:10.1038/s41467-020-16308-3)
Supplement: Supplementary file 3 — Reporting Summary [file 41467_2020_16308_MOESM3_ESM.pdf]

nature research

Corresponding author(s): Stephen Freedman
Last updated by author(s): Apr 10, 2020

Reporting Summary

Nature Research wishes to improve the reproducibility of the work that we publish. This form provides structure for consistency and transparency in reporting. For further information on Nature Research policies, see Authors & Referees and the Editorial Policy Checklist .

Statistics

For all statistical analyses, confirm that the following items are present in the figure legend, table legend, main text, or Methods section.

n/a	Confirmed	
☐	☒	The exact sample size (n) for each experimental group/condition, given as a discrete number and unit of measurement
☒	☐	A statement on whether measurements were taken from distinct samples or whether the same sample was measured repeatedly
☐	☒	The statistical test(s) used AND whether they are one- or two-sided<br>*Only common tests should be described solely by name; describe more complex techniques in the Methods section.*
☐	☒	A description of all covariates tested
☐	☒	A description of any assumptions or corrections, such as tests of normality and adjustment for multiple comparisons
☐	☒	A full description of the statistical parameters including central tendency (e.g. means) or other basic estimates (e.g. regression coefficient) AND variation (e.g. standard deviation) or associated estimates of uncertainty (e.g. confidence intervals)
☐	☒	For null hypothesis testing, the test statistic (e.g. F, t, r) with confidence intervals, effect sizes, degrees of freedom and P value noted<br>*Give P values as exact values whenever suitable.*
☒	☐	For Bayesian analysis, information on the choice of priors and Markov chain Monte Carlo settings
☒	☐	For hierarchical and complex designs, identification of the appropriate level for tests and full reporting of outcomes
☒	☐	Estimates of effect sizes (e.g. Cohen's d, Pearson's r), indicating how they were calculated

Our web collection on statistics for biologists contains articles on many of the points above.

Software and code

Policy information about availability of computer code

Data collection	PCR amplification profiles were analyzed using Sequence Detection Software version 1.0. To sequentially assign children to probiotic or placebo, we employed Randomize.net, version 3.6.
Data analysis	N/A

For manuscripts utilizing custom algorithms or software that are central to the research but not yet described in published literature, software must be made available to editors/reviewers. We strongly encourage code deposition in a community repository (e.g. GitHub). See the Nature Research guidelines for submitting code & software for further information.

Data

Policy information about availability of data

All manuscripts must include a data availability statement. This statement should provide the following information, where applicable:
- Accession codes, unique identifiers, or web links for publicly available datasets
- A list of figures that have associated raw data
- A description of any restrictions on data availability

The full study protocol and the datasets, which includes all data fields reported in this study, are available, following manuscript publication, upon request from the corresponding author (Dr. Stephen Freedman, Stephen.Freedman@AlbertaHealthServices.ca), following the provision of ethics approval. The source data underlying Figs 2 and 3a–c are provided as a Source Data file.

Field-specific reporting

Please select the one below that is the best fit for your research. If you are not sure, read the appropriate sections before making your selection.

Life sciences study design

All studies must disclose on these points even when the disclosure is negative.

Sample size	For the primary outcome we a priori anticipated that follow-up would be complete for 90% of clinical trial participants (N=797) and all of these participants would thus have follow-up MVS scores. Based on North American15,48,49 data, we assumed the following pathogen distributions: ~50% viral (N=399), ~40% unidentified (N=318), and ~10% bacterial (N=80). Given our 1:1 probiotic:placebo allocation ratio, we anticipated a minimum of 40 participants per arm in the smallest group.50 The proposed minimum clinically important pathogen-group and pathogen-specific MVS difference of means were based on the natural history of disease,35 and the proposed benefits associated with probiotic administration (Supplementary Table 1). Power calculations assumed 40 subjects in each study arm pathogen group (i.e. probiotic and placebo virus, bacteria, and unidentified) and a standard deviation (SD) of 3.135 around the MVS score point estimates. Based on our proposed effect sizes power calculations were > 80%, assuming a minimum of 40 paired specimens for each pathogen group comparison. Similar power was present when the analysis was repeated looking at specific viral etiologies (smallest cell = 39).
Data exclusions	No data were excluded.
Replication	Usually, experiment replication is performed to confirm data accuracy. In our current study we did not replicate the viral load experiments as we are very confident in our results as we employed two tiers of testing. The initial test, the Luminex Gastrointestinal Pathogen Panel, was a qualitative test (i.e. positive/negative). This test was employed to determine the presence of a pathogen (i.e. norovirus, rotavirus and adenovirus). Subsequently, positive cases underwent qPCR quantifcation. In order to eliminate the inter-run variation, three samples collected (day 0, day 5 and day 28) from each patient were tested in the same experiment. Thus, we are confident in the precision and accuracy of our data.
Randomization	To sequentially assign children to probiotic or placebo, we employed a random-number–generating software, accessed through www.randomize.net, which was programed to use block sizes of 4 and 6, stratified according to site. The random allocation sequence was generated by the research pharmacy at the coordinating center. Participants were enrolled by research nurses or assistants at each site who provided caregiver with the allocation assignment. Participants and their parents or guardians, trial and clinical staff, and specimen and data analysts remained blinded to treatment assignment through the use of a placebo that was identical in appearance, smell, and weight to the intervention agent (i.e. probiotic).
Blinding	Participants and their parents or guardians, trial and clinical staff, and specimen and data analysts remained blinded to treatment assignment through the use of a placebo that was identical in appearance, smell, and weight to the intervention agent (i.e. probiotic). All stool tests were conducted blinded to treatment allocation and clinical symptoms.

Reporting for specific materials, systems and methods

We require information from authors about some types of materials, experimental systems and methods used in many studies. Here, indicate whether each material, system or method listed is relevant to your study. If you are not sure if a list item applies to your research, read the appropriate section before selecting a response.

Materials & experimental systems

n/a	Involved in the study	
☒	☐	Antibodies
☒	☐	Eukaryotic cell lines
☒	☐	Palaeontology
☒	☐	Animals and other organisms
☐	☒	Human research participants
☐	☒	Clinical data

Methods

n/a	Involved in the study	
☒	☐	ChIP-seq
☒	☐	Flow cytometry
☒	☐	MRI-based neuroimaging

Human research participants

Policy information about studies involving human research participants

Population characteristics	Patients were aged 3 to 48 months with AGE presenting for ED care, were randomly assigned, in a 1:1 ratio, to receive 4.0×109 colony forming units of a L. rhamnosus R0011 and L. helveticus R0052 (95:5 ratio) probiotic preparation or matching placebo twice daily for 5 days, in addition to usual care. Eligible children had ≥3 episodes of diarrhea in a 24-hour period, and had vomiting or diarrhea for < 72 hours. All children were evaluated by a physician who assigned a diagnosis of acute gastroenteritis. Children were excluded if they or a person living in their household had a central venous line, structural heart disease, were immunocompromised, or were receiving immunosuppressive therapy. Children who presented with a history of oral or gastrointestinal surgery within the preceding 7 days, blood in their vomit or stool, bilious vomiting, a chronic intestinal disorder, pancreatic insufficiency, probiotic use in the preceding 14 days, soy allergy, and an inability to complete follow-up were also excluded. 229 (56.1%) of study participants in the probiotic group, and 235 (57.6%) in the placebo group, were males.
Recruitment	Participants were enrolled by research nurses or assistants at each site who provided caregiver with the allocation assignment. Given that allocation assignment was only performed by a web-based system (www.randomize.net) following enrollment (i.e. families, physicians, and research personnel were completely blinded to allocation. Thus, it is highly unlikley that the probability of a ptient being enrolled would have depended on the probability of them being assigned to a particular treatment group.
Ethics oversight	The protocol was approved by the research ethics boards at each of the six participating Canadian tertiary care pediatric centers located in Calgary (Conjoint Health Research Ethics Board), London (Western University Health Sciences Research Ethics Board), Toronto (SickKids Research Ethics Board), Ottawa (Children's Hospital of Eastern Ontario Research Ethics Board), Montreal (Comité d'éthique de la recherche du CHU Sainte-Justine) and Halifax (IWK Research Ethics Board), in Canada.

Note that full information on the approval of the study protocol must also be provided in the manuscript.

Clinical data

Policy information about clinical studies

All manuscripts should comply with the ICMJEguidelines for publication of clinical research and a completedCONSORT checklist must be included with all submissions.

Clinical trial registration	Registered at www.clinicaltrials.gov: NCT01853124
Study protocol	It has been submitted to the journal and is published at: https://www.nejm.org/doi/suppl/10.1056/NEJMoa1802597/suppl_file/nejmoa1802597_protocol.pdf
Data collection	Children were enroled between November 5, 2013, and April 7, 2017 in Calgary, London, Toronto, Ottawa, Montreal and Halifax. Participant caregivers provided baseline (Day 0) data while in the emergency departments of the respective institutions Calgary (Alberta Children's Hospital; University of Calgary) , London (London Health Sciences Centre; University of Western Ontario), Toronto (The Hospital for Sick Children; University of Toronto), Ottawa (Children's Hospital of Eastern Ontario; University of Ottawa), Montreal (Centre Hospitalier Ste. Justine; Universite de Montreal) and Halifax (IWK Hospital; Dalhousie University).
Outcomes	The primary objective was to determine if a 5-day probiotic treatment course administered to children with AGE resulted in pathogen-specific clinical benefits quantified using the validated and widely-used33 Modified Vesikari Scale (MVS) score.34,35 Secondary objectives identified a priori included 1) assessing if probiotic administration resulted in a greater reduction in stool pathogen load compared with placebo; and determining the relationship between 2) correlating baseline (Day 0) stool pathogen load and baseline MVS score, and 3) Day 5 stool pathogen load and the follow-up MVS score.
